# Thwarting the Divine Will

**Published:** 1891-08

**Authors:** 


### THWARTING THE DIVINE WILL.

Mr. J. J. Dukes, a prominent citizen of Americus, Ga., has got into trouble. Not long since he had a neat home built, and had it fitted up in a nice and comfortable manner. As is usual, he had hardly completed his building before the irrepressible lightning rod agent put in his appearance, and with suave manner and smooth language brought countless reasons why protection should be sought from the electrical element. Mr. Dukes finally decided to accept the offer, and soon had the little steel points reaching out from the house.

He has for years been a member in good standing of the Hardshell church just north of this place, and having the work done, continued to attend faithfully and perform his duties as a member. His co-workers in the church, however, viewed with great concern the heretical performance above mentioned, and many of the good sisters and brethren labored with him to have the rod removed. The watchful pastor talked and prayed with the wanderer from his flock, but all to no purpose. Mr. Dukes couldn't see the harm and the rods remained.

Finally the matter had gone as far as the church, as a body, could permit, and a meeting was held to discuss it. They sent committee after committee to the wayward brother, but all to no purpose, and then it was decided to make a test case of the matter. A committee was sent to him, a short time since, to inform him that early in May he would be tried by the church for going contrary to the doctrine he professed.

In this case they hold that the will of God has been interfered with, and thus the brother has done an action for which he must answer to the church.

